# Tobacco-free Nicotine Pouch Use in Great Britain: A Representative Population Survey 2020–2021

**DOI:** 10.1093/ntr/ntac099

**Published:** 2022-04-13

**Authors:** Harry Tattan-Birch, Sarah E Jackson, Martin Dockrell, Jamie Brown

**Affiliations:** Department of Behavioural Science and Health, University College London, London, UK; SPECTRUM Consortium, UK; Department of Behavioural Science and Health, University College London, London, UK; SPECTRUM Consortium, UK; Addictions and Inclusion, Office for Health Improvement and Disparities, London, UK; Department of Behavioural Science and Health, University College London, London, UK; SPECTRUM Consortium, UK

## Abstract

**Introduction:**

Tobacco-free nicotine pouches are products that are placed between the lip and gum, where they deliver nicotine to users. Little is known about nicotine pouch use in Great Britain since they entered the market in 2019.

**Methods:**

Data came from a monthly representative survey of the adult (≥18 years) population in Great Britain (England, Scotland, and Wales) between November 2020 and October 2021 (*n* = 25 698). We estimated the weighted prevalence of pouch use, overall and stratified by demographics, smoking status, and other nicotine use.

**Results:**

Nicotine pouch use was rare among adults, with a weighted prevalence of just 0.26% (95% compatibility interval [CI] = 0.19–0.35). Prevalence doubled from November 2020 to October 2021 (0.14%–0.32%; prevalence ratio [PR] = 2.22, 95% CI = 1.33–3.70). Pouch use was over four times more common among men than women (0.42% vs. 0.09%; PR = 4.55, 95% CI = 2.27–9.09) but less common in older age groups (*p* < .001). Pouch use was more prevalent among current smokers (0.87%; PR = 13.60, 95% CI = 5.46–33.89), recent former smokers (0.97%; PR = 15.21, 95% CI = 4.03–57.42), and long-term (>1 year) former smokers (0.24%; PR = 3.71, 95% CI = 1.36–10.15), compared with never smokers (0.06%). Prevalence was also elevated among e-cigarette (1.64% vs. 0.15%; PR = 10.59, 95% CI = 5.74–19.52) and nicotine replacement therapy users (2.02% vs. 0.21%; PR = 9.75, 95% CI = 4.64–20.49).

**Conclusions:**

One in 400 adults in Great Britain use nicotine pouches, but the prevalence increased from 2020 to 2021.

**Implications:**

Tobacco-free nicotine pouches were introduced to the market in Great Britain in 2019. We found that while pouch use is currently rare in Great Britain, these products have become more popular over time. Pouch use is largely concentrated among younger and middle-aged men who use other nicotine products and have a history of smoking. Continued monitoring of nicotine pouch use is needed.

## Introduction

The global nicotine market is volatile, with many new products launching each year.^1–3^ One recent innovation is the tobacco-free oral nicotine pouch.^4^ These nicotine pouches are used in the same way as Swedish snus, placed between the lip and gum where they rapidly and effectively deliver nicotine.^5^ Unlike snus, they contain nicotine extract rather than processed tobacco leaf and are thus exempt from the EU and United Kingdom ban on oral tobacco.^6^ Nicotine pouches were first introduced to European markets outside of Scandinavia in 2019.^7^ All major tobacco companies now sell them, with popular brands including Zyn, Velo, and Nordic Spirit.^8^

Little is known about the prevalence of nicotine pouch use globally, with research to date coming from an online questionnaire in the Netherlands^9^ and three non-representative/selective samples in the United Kingdom, North America, and Australia.^10–12^ Knowing how many people use nicotine pouches, and tracking how this is changing over time, is necessary to determine the scale to which these products could affect public health, either positively—by encouraging cigarette smokers to switch to a lower risk product, or negatively—by, for example, attracting people who would otherwise avoid nicotine entirely. Using data from a representative population survey, we aimed to estimate the prevalence of nicotine pouch use among adults in Great Britain, assessing how use differs by age, gender, social grade, country of residence, smoking status, and use of other nicotine products.

## Methods

### Design

Data were used from the Smoking Toolkit Study (STS), a monthly cross-sectional survey that recruits a representative sample of adults (≥18 years) in Great Britain (England, Scotland, and Wales). Sampling methods are described in detail elsewhere.^13,14^ Briefly, Great Britain is split into output areas, each with ~300 households. These output areas are stratified by region and demographic characteristics, before being randomly selected for inclusion on the interview list. Interviews are conducted in these selected areas until quotas based on working status, age, and gender are met. Survey weights are constructed with raking to adjust data so that the sample matches the demographic profile of Great Britain. This profile is determined each month by combining data from the 2011 UK Census, the Office for National Statistics mid-year estimates, and the annual National Readership Survey. Comparisons with other national surveys and with cigarette sales data show that the STS provides estimates that are representative with respect to key demographic and smoking-related variables.^13,15^ Data on the use of other nicotine and tobacco products are published regularly at https://smokinginengland.info/.

### Participants

This analysis included participants who completed telephone interviews between November 2020, the first wave to ask about nicotine pouch use, and October 2021, the latest available data at the time of analysis. Ethical approval was provided by the UCL Research Ethics Committee (0498/001). Participants provided informed consent to take part in the study, and all methods were carried out in accordance with relevant regulations.

### Measures

Nicotine pouch use was ascertained by asking participants whether they currently use “tobacco-free nicotine pouch/pod or ‘white pouches’ that you place on your gum (eg, Zyn, On!, Nordic Spirit, Dryft/Velo, Lyft, Skruf)”. Demographic variables were age, gender, occupational social grade (NRS classification of AB, C1, C2, D, and E), and country of residence (England, Scotland, and Wales). Smoking status (current, recent [≤1 year] former, long-term [>1 year] former, and never smoker), current e-cigarette use (vaping), and current nicotine replacement therapy (NRT) use were also measured.

### Statistical Analysis

We calculated the number and percentage of participants who used nicotine pouches. Log-binomial regression was used to estimate the weighted prevalence of nicotine pouch use, both overall and stratified by demographic characteristics, smoking status, and use of other nicotine products. One-way associations between nicotine pouch use and each of these variables were reported as prevalence ratios (PRs) with 95% compatibility (“confidence”) intervals (95% CIs).^16^ To measure time trends in prevalence, we ran a log-binomial regression with survey month modeled using restricted cubic splines with three knots placed at quantiles, thus allowing for non-linear relationships.^17^ The same method was used to model trends in prevalence across ages.

## Results

Of the 25 698 adults surveyed in Great Britain from November 2020 to October 2021, 54 (0.21%) reported currently using nicotine pouches. After applying survey weights, the estimated prevalence of nicotine pouch use was 0.26% (95% CI = 0.19–0.35). Pouch use became more common over time, increasing from 0.14% in November 2020 to 0.32% in October 2021 (PR = 2.22, 95% CI = 1.33–3.70)—as shown in Supplementary Figure 1.

Table 1 shows the weighted prevalence of pouch use among different demographic groups. Prevalence was similar in England (0.25%), Scotland (0.32%), and Wales (0.25%). There were gender differences, with men being over four times as likely to use nicotine pouches as women (0.42% vs. 0.09%; PR = 4.55, 95% CI = 2.27–9.09). Prevalence of nicotine pouch use was lower in older than middle-aged and young adults, as shown in Supplementary Figure 2 (0.06% for ≥65-year-olds compared with 0.49% for 16- to 24-year-olds and 0.54% for 35- to 44-year-olds; *p* < .001). It is unclear how pouch use differs by occupational social grade, a measure of socioeconomic position, because of the low numbers of pouch users (eg, 3 users in social grade E) surveyed in each occupational group (*p* = .083).

**Table 1.** Nicotine Pouch Use Across Demographics in Great Britain

	Current nicotine pouch use				*p* ^a^
	Not current user, *N* (column %)	Current user, *N* (column %)	Prevalence, row % (95% CI)	Prevalence ratio, PR (95% CI)	
Overall	25 577	66	0.26 (0.19–0.35)		
Social grade					.083
AB (most advantaged)	7060 (27.6%)	15 (23.5%)	0.22 (0.12–0.40)	Ref	
C1	6782 (26.5%)	11 (16.8%)	0.16 (0.10–0.28)	0.74 (0.33–1.67)	
C2	5436 (21.3%)	22 (33.2%)	0.40 (0.22–0.71)	1.83 (0.79–4.23)	
D	3830 (15.0%)	15 (22.3%)	0.38 (0.19–0.79)	1.75 (0.68–4.50)	
E (least advantaged)	2468 (9.7%)	3 (4.2%)	0.11 (0.04–0.34)	0.52 (0.15–1.80)	
Age (years)					<.001
18–24	2651 (10.4%)	13 (20.0%)	0.49 (0.23–1.08)	Ref	
25–34	4362 (17.1%)	13 (19.2%)	0.29 (0.15–0.56)	0.59 (0.21–1.63)	
35–44	4094 (16.0%)	22 (33.7%)	0.54 (0.32–0.91)	1.09 (0.43–2.80)	
45–54	4399 (17.2%)	8 (12.7%)	0.19 (0.08–0.43)	0.39 (0.12–1.19)	
55–64	4004 (15.7%)	6 (9.0%)	0.15 (0.06–0.36)	0.30 (0.09–0.98)	
65+	6028 (23.6%)	3 (5.3%)	0.06 (0.02–0.18)	0.12 (0.03–0.47)	
Gender^b^					<.001
Women	12 999 (50.8%)	12 (18.8%)	0.09 (0.05–0.17)	Ref	
Men	12 578 (49.2%)	53 (81.2%)	0.42 (0.30–0.60)	4.55 (2.27–9.09)	
Country					.774
England^c^	22 049 (86.2%)	55 (84.1%)	0.25 (0.18–0.36)	Ref	
Scotland	2276 (8.9%)	7 (11.2%)	0.32 (0.18–0.58)	1.29 (0.65–2.57)	
Wales	1252 (4.9%)	3 (4.7%)	0.25 (0.10–0.63)	0.99 (0.36–2.69)	
Smoking status					<.001
Never	14 809 (57.9%)	9 (14.4%)	0.06 (0.03–0.14)	Ref	
Long-term (>1 year) former	6051 (23.7%)	14 (21.9%)	0.24 (0.13–0.43)	3.71 (1.36–10.15)	
Recent (≤1 year) former	557 (2.2%)	5 (8.3%)	0.97 (0.34–2.78)	15.21 (4.03–57.42)	
Current	4160 (16.3%)	36 (55.4%)	0.87 (0.57–1.32)	13.60 (5.46–33.89)	
E-cigarette use					<.001
No	23 841 (93.2%)	37 (56.1%)	0.15 (0.10–0.23)	Ref	
Yes	1736 (6.8%)	29 (43.9%)	1.64 (1.04–2.58)	10.59 (5.74–19.52)	
NRT use					<.001
No	24 888 (97.3%)	52 (78.4%)	0.21 (0.15–0.29)	Ref	
Yes	689 (2.7%)	14 (21.6%)	2.02 (1.04–3.90)	9.75 (4.64–20.49)	

Abbreviations: CI = compatibility (“confidence”) interval; NRT = nicotine replacement therapy; PR = prevalence ratio.

*p*-values ascertained using likelihood ratio tests against an intercept-only model.

All nicotine pouch users identified as either a man or woman.

Prevalence estimates across regions of England are shown in Supplementary Table 1.

Pouch use was more common among current smokers (0.87%; PR = 13.60, 95% CI = 5.46–33.89), recent former smokers (0.97%; PR = 15.21, 95% CI = 4.03–57.42), and long-term (>1 year) former smokers (0.24%; PR = 3.71, 95% CI = 1.36–10.15), compared with never smokers (0.06%). Prevalence was also elevated among people who were currently using e-cigarettes (1.64% vs. 0.15%; PR = 10.59, 95% CI = 5.74–19.52) or NRT (2.02% vs. 0.21%; PR = 9.75, 95% CI = 4.64–20.49).

## Discussion

Nicotine pouch use is rare in Great Britain, with just one in every 400 adults currently using these products. This equates to a total of 130 000 (95% CI = 100 000–180 000) nicotine pouch users across Great Britain—110 000 (80 000–160 000) in England, 14 000 (8000–25 000) in Scotland, and 6000 (2500–16 000) in Wales.^18^ Prevalence is increasing over time, with twice as many people using pouches in October 2021 than in November 2020.

Prevalence is higher among men than women and among young or middle-aged adults than older adults. These results are consistent with data from online surveys in the Netherlands,^9^ the United Kingdom,^10^ Australia, Canada, and the United States,^12^ which also found a relatively low prevalence of nicotine pouch use in women and older adults. They also mirror historic gender differences in the use of snus (tobacco-containing pouches) in Nordic countries.^19^

We found that nicotine pouch use is concentrated among adults who use other nicotine products and have a history of smoking. This means pouches are currently unlikely to be attracting substantial numbers of people who would otherwise avoid nicotine entirely in Great Britain. Nonetheless, it could take years for nicotine pouches to achieve widespread popularity. It is possible that, following the diffusion of innovations,^20^ the “early adopters” of nicotine pouches have different characteristics than the majority of users once the market reaches saturation. For instance, early adopters of e-cigarettes may have come from more advantaged groups than later users.^21–23^ Therefore, continued monitoring of the characteristics of people using nicotine pouches is needed.

Our study benefits from using a representative survey of the population in Great Britain, collecting detailed data on demographics and nicotine use. The repeat cross-sectional design allows us to track changes over time—which was useful for this study in examining changes from 2020 to 2021 but will also be important for continued monitoring beyond this report. Limitations include the absence of a measure of former nicotine pouch use, which meant we could only examine the percentage of people who were currently using nicotine pouches when interviewed, not the percentage who had ever tried them. There was also no measure of whether pouches were the first nicotine product a person used, but as pouches were only introduced to Great Britain in 2019, it is unlikely that participants tried pouches before cigarettes, e-cigarettes, or NRT. While it is not clear what caused the prevalence of pouch use to increase over time, the trend is unlikely to be explained by factors associated with COVID-19 because the pandemic was present throughout the entire period studied.

In conclusion, while nicotine pouch use is currently uncommon in Great Britain, it grew between 2020 and 2021. Pouch use is largely concentrated among younger and middle-aged men who also use other nicotine products and have a history of smoking.

## Supplementary Material

A Contributorship Form detailing each author’s specific involvement with this content, as well as any supplementary data, are available online at https://academic.oup.com/ntr.

ntac099_suppl_Supplementary_Material

ntac099_suppl_Supplementary_Taxonomy-Form

## Data Availability

Data are available on reasonable request to the corresponding author.

## Registration

The analysis plan was registered a priori (https://osf.io/asnqy/).
