# Supplementary material for: Tobacco-free Nicotine Pouch Use in Great Britain: A Representative Population Survey 2020–2021
Source: Nicotine Tob Res. 2022 Apr 13;24(9):1509–12. doi: 10.1093/ntr/ntac099 (PMC9356773; doi:10.1093/ntr/ntac099)
Supplement: ntac099_suppl_Supplementary_Material [file ntac099_suppl_supplementary_material.docx]

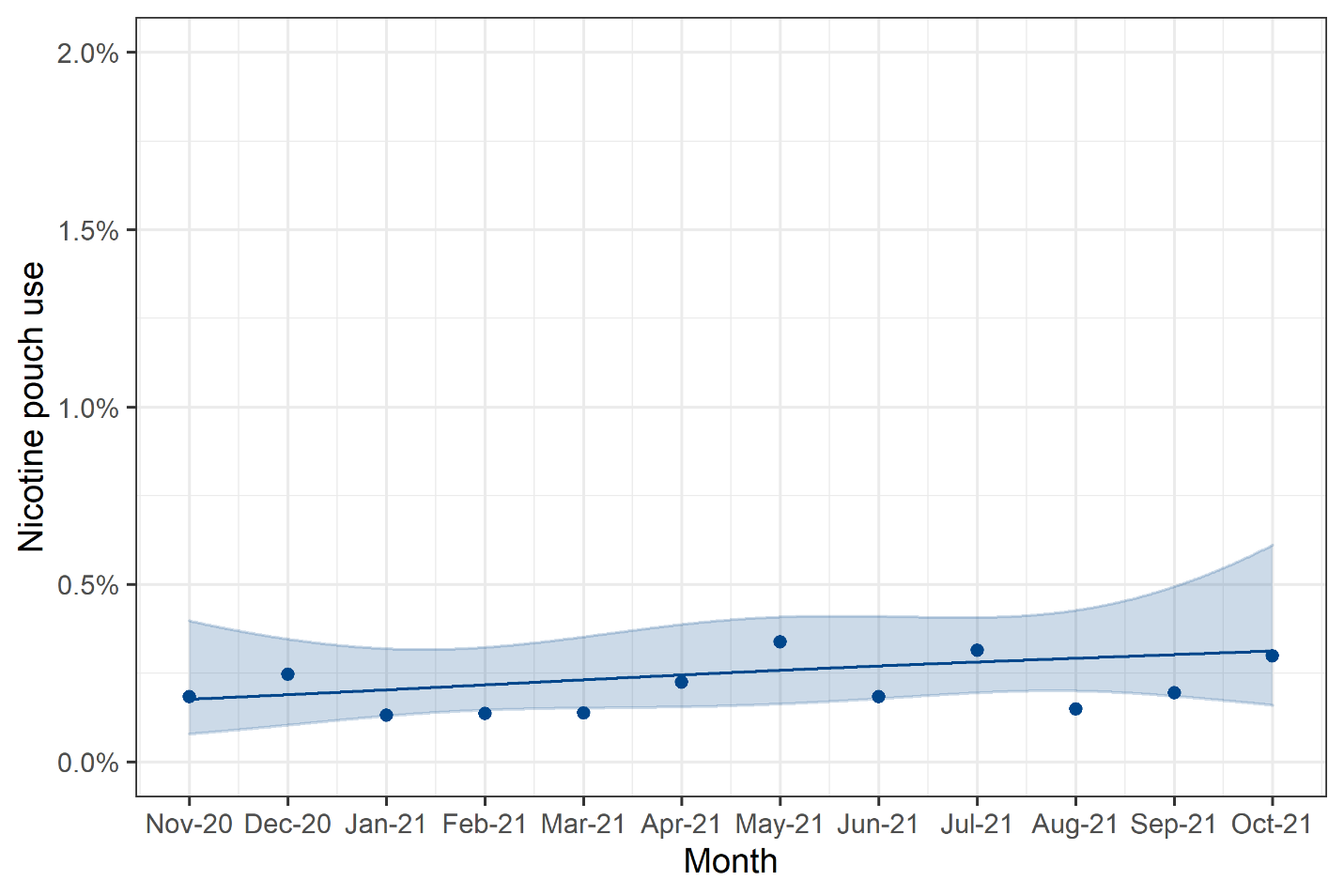
**Supplementary figure 1: Trends in prevalence of nicotine pouch use in Great Britain from November 2020 to October 2021.** The line represents fitted values from weighted log-binomial regression, using natural cubic splines (knots=3) to model month. Shaded bands represent 95% CIs. Points show the unweighted percentage of participants who reported nicotine pouch use in each month.


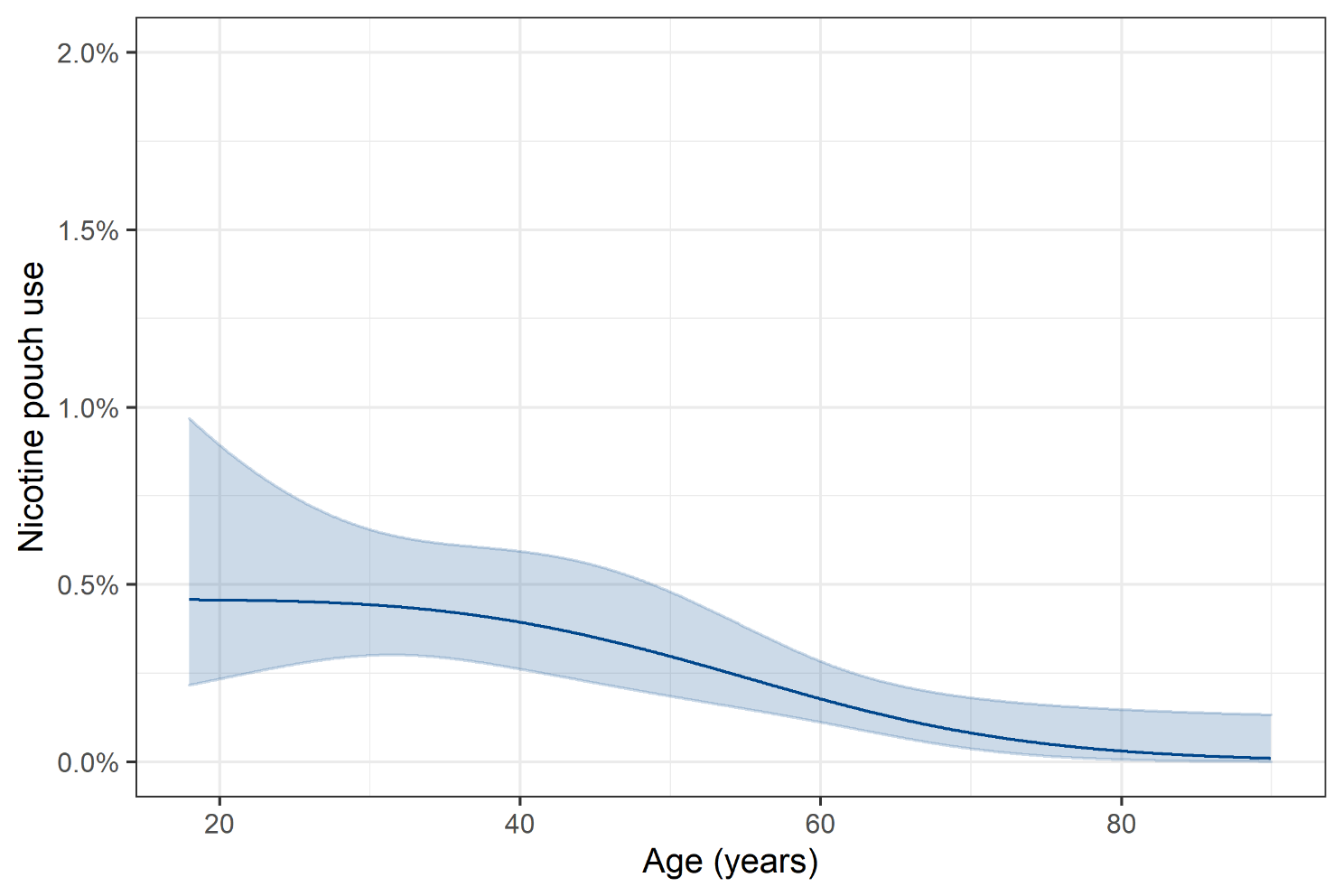
**Supplementary figure 2: Prevalence of nicotine pouch use by age in Great Britain.** The line represents fitted values from weighted log-binomial regression, using natural cubic splines (knots=3) to model age. Shaded bands represent 95% CIs. Points along the x-axis represent nicotine pouch users of a given age, with jitter applied for clarity.

**Supplementary Table 1: Nicotine pouch use across the regions of England.**

|  | **Current nicotine pouch use** | | | |  |
| --- | --- | --- | --- | --- | --- |
|  | **No**,  *N* (%) | **Yes**,  *N* (%) | **Prevalence,**  % (95% CI) | **Prevalence Ratio,**  (95% CI) | ***p* ^†^** |
| **Region** |  |  |  |  | .831 |
| North East | 1,078 (4.2%) | 4 (6.7%) | 0.41 (0.13-1.31) | Ref |  |
| North West | 2,870 (11.2%) | 12 (18.8%) | 0.43 (0.20-0.92) | 1.00 (0.25-4.10) |  |
| Yorkshire and the Humber | 2,138 (8.4%) | 6 (9.9%) | 0.30 (0.09-0.97) | 0.69 (0.13-3.57) |  |
| East Midlands | 1,908 (7.5%) | 4 (6.4%) | 0.22 (0.07-0.68) | 0.58 (0.11-2.99) |  |
| West Midlands | 2,313 (9.0%) | 5 (7.4%) | 0.21 (0.08-0.55) | 0.50 (0.11-2.27) |  |
| East of England | 2,457 (9.6%) | 9 (14.3%) | 0.38 (0.15-0.99) | 0.90 (0.20-4.12) |  |
| London | 3,394 (13.3%) | 4 (6.8%) | 0.13 (0.05-0.36) | 0.37 (0.08-1.79) |  |
| South East | 3,614 (14.1%) | 6 (8.5%) | 0.15 (0.06-0.43) | 0.39 (0.08-1.85) |  |
| South West | 2,279 (8.9%) | 3 (5.3%) | 0.15 (0.04-0.61) | 0.40 (0.06-2.51) |  |

^†^ *p*-value ascertained using likelihood ratio test against an intercept only model.
